# Dental Ethics

**Published:** 1866-05

**Authors:** M. S. Dean

**Affiliations:** Chicago, Ill.


### DENTAL ETHICS.

BY DR. M. S. DEAN, CHICAGO, ILL.

Read before the Chicago Dental Society.

Mr. President, and Gentlemen. With the exception perhaps of the Sacred Ministry, there is no profession in which a higher standard of morality is imperatively necessary than that of *Dentistry*; and, unless it be that of medicine, there is no profession in which the practioner is beset more frequently with temptation to swerve from the line of strict integrity, and in which there are oftener delicate and difficult questions of duty presenting themselves, which require judgment self-denial and the aid of sound morality.

The object of this paper is to suggest some comprehensible rules, whereby the conduct of our professional life should be guided and governed; and as the writer will be able merely to touch upon a few points which first present themselves to his view, he cannot be expected to arrive at any very clear and definite rules, which will apply to each individual case that may arise in practice. But he hopes, at least, that it may be the means of eliciting the views of the society, which will result in the establishment of a code of *Dental Ethics*, founded on high moral principle.

Professional skill is due to the patient from the dentist; professional skill *honestly* directed. If the teeth are not skillfully treated by the operator, the patient is robbed, not only of his money, but, oftentimes, of his personal beauty, his comfort, and those living organs which science and art are unable to restore. The dentist, therefore, should not only be skilled in his art, but should direct that skill with judgment, care, and fidelity. These qualities are the more essential, because his operations are commonly of such a nature that his patron has no means of judging either their quality or their value. If he employ an artist to paint his portrait, the living features must be faithfully portrayed on the canvas, or the work is pronounced by himself and friends, as a worthless caricature. He is also, with tolerable accuracy, enabled to determine the character and worth of mechanic labors, and the handiwork of the artisan. But when he employs a dentist, he employs a man who is generally the sole arbiter, as to both the quality and value of his work, and must rely, wholly upon his *skill* and *integrity*; and although it is impossible for every dentist to acquire the first to an equal extent, the latter is fully within the control, and should be possessed by each, in an eminent degree.

These remarks cannot apply, with equal force, to what is termed the mechanical branch of our profession; although in *this*, our patrons rely almost solely on our judgement, mechanical and artistic skill, yet this work goes forth, to be submitted to the test of criticism from the most scrutinizing eyes. But if our labors in this branch of dentistry, prove to be worthless, we have only imposed on the confidence of our patients, and defrauded them of a little money; in the other case we have added, to both of these the crime of destroying the priceless living dentures. How important, then, that we possess not only skill, but that sound moral principle, and strict integrity, should guide our professional conduct.

As before intimated the dentist is not unfrequently beset with temptation to deviate from the strict line of duty; sometimes from a genuine sympathy for his suffering and sensitive patient; Sometimes from his insulting language, and ungovernable action; sometimes from a consciousness that his best efforts will not be fairly requited nor fully appreciated; and sometimes, from his own lack of time, or want of energy, or from various other causes, he is tempted to perform his operations in a careless, hasty, or indifferent manner. When thus assailed by temptation, the dentist should gird himsef in the bright armor of *Fidelity*, and his instruments, like the spear of the guardian angel of Paradise, should be "touched with celestial temper."

Skill and integrity, it has been remarked, are due to the patient from the dentist; so likewise are patience, kindliness, and respectful courtesy. The dentist should not be a man of vulgar, or clownish manner, but a man of-good breeding, politeness, and civil deportment. *For his profession is the offspring of refinement*; and from its very nature, he is necessarily brought into more intimate social relation with the more polished and intelligent classes of society than are the members of any other profession. Hence, he should not only be skillful, but a man of culture and refinement.

His reception rooms should be comfortable, appropriately furnished, and cheerful in their appearance; with a few books and drawings, perhaps, to amuse and divert the attention of his patients in waiting, and to prevent them from suffering those imaginary tortures, which are generally more excruciating than they ever realize. And *here*, as also in the operating room, there should be no display of anatomical specimens or drawings to shock the sensitive mind; nor show-cases containing exhibition of his handiwork. The operating room should be convenient, and devoid of all ostentatious show of instruments; for that day has gone by, when a brilliant display of pearl and and polished steel, are taken as evidences of the skillfulness of the dentist. His instruments, however, should be kept in perfect order, and so arranged that the one he needs may be quickly selected, without that confusion, which would be the result of a want of system. His patient should not be nauseated by a foul and odorous spittoon, nor rusty instruments perfumed with creosote, and bespattered with blood, nor by the uncleanly hands of the operator. But spittoons, napkins, instruments, and whatever is used in and about the mouth, should be scrupulously clean. While the dentist *generally* grows more careful in regard to the quality of his operations, he is too apt to become careless and slovenly in the minor details, which are most essential to the comfort of his patient, while these operations are being performed.

He should receive his patients with polite attention, but not with obsequiousness, nor excessive complaisance. His manners, should be easy and unconstrained, but not too familiar; and, bearing in mind the delicate and sensitive structure which he is about to treat, and perhaps the nervous temperament of his patient, he should endeavor to allay her fears, and proceed with as much care and gentleness, as is consistent with a thorough treatment of the case. His patient should be made to understand that he is not reckless in the use of the pointed steel, nor totally regardless of the amount of pain he may inflict. To do this, he need not be profuse in his protestations of sympathy, for too many words are often worse than none. Nor should his sympathies ever be allowed to arrest his hand, in the faithful performance of its duty.

Equanimity and self-possession are also qualities of unspeakable value to the dentist; although his task is generally a wearisome and perplexing one, and his patience is often sorely tried, by the unguarded and inconsiderate speech or actions of his patient, while he is taxing his best energies in his behalf, yet it is highly important that his temper should be always even, and that he carefully repress everything like excitabillity, or irritabillity. For if passion be allowed to prevail, his judgement and dignity are dethroned, and he degrades himself in his own esteem, and in the eyes of his patient. And, while he should always be firm, without being obstinate, there are times when both his manhood and his duty require him to treat his patient, not with anger or petulance, but with *moral severity.* There is however *less* danger of our allowing these unpleasant occasions to pass unnoticed and unrebuked, than there is of our anticipating them.

The young dentist is naturally anxious to do a large business at once, and is to apt to resort to the many means at hand to gain notoriety, and attract public attention, with the view of bringing patients to his office. The newspapers teem with his advertisements, setting forth his passing skill, in glowing terms, and the wonderful cheapness of his painless operations, and circulars of the same import steal under the door of every shop and dwelling. At the entrance of his office may be found perhaps, exhibitions of his art or ingenious contrivances to attract the attention of, and arrest the passer-by. Thus, from a desire of commanding immediately, a large and lucrative practice, he lowers his own dignity; and hangs as a dead weight upon the skirts of his profession. A less mortifying course, and one far more satisfactory in its final result, and honorable to the profession which he has adopted, would be for him to confine himself, generally, to his office, even if it should require some self-denial and privation, and make himself a thorough master of his art; devoting his leisure time to study and experimental practice. And in the mean time, the business that comes in, at first perhaps, slowly, let him treat with great care and fidelity. Let him possess habits of neatness, punctuality and candor, towards his patients, and strict honor towards his professional brethren. And it may be safely predicted that his business will grow as fast, as it is good for him that it should grow ; while he gradually becomes able to sustain a large practice without being bewildered or overwhelmed.

There has been in ours, more than in that of any other profession, except that of medicine, a disposition on the part of its members to disparage the skill of their cotemporaries in practice. And, although that spirit still exists to a considerable extent, though there are some yet who are either so conceited, or so selfish as to *seem* to believe that skill can reside in no other hand than their own, their number is few and rapidly becoming less. These by their unjust criticism, and sneering manner, injure the profession, and wrong its members, without doing themselves any permanent benefit. But there are cases which come within the observation of every dentist, that show either the lack of skill or honesty or both, on the part of the practioner, and which are totally inadequate to accomplish the object for which they were designed; teeth inserted which are unnatural in appearance uncomfortable, and useless; fillings, which only serve to lull the patient into fancied security in regard to his teeth, while decay is insidiously stealing its way, rapidly, towards the nerve pulp. When the person who is the subject of these, or other unsuccessful operations, seeks our advice and aid, it is undoubtedly our duty, though it be a painful one, to point out their defect, and suggest the remedy. And if we should find the major part of this work to have been skillfully performed, the patient should be made acquainted with that fact. And if there should be any possible reason for believing that some circumstances, not within the control of the dentist, might have prevented him from doing *all* of his work skillfully, we should not condemn the operator, but the result of his operations. For who is there among us with such unbounded confidence, and with so little experience, that he dares to assert that all of *his* operations will prove to be good and substantial? Whilst we should be exceedingly careful not to unjustly disparage the works of others, there is a class of operations which we sometimes meet with, that shows incontestably the want of skill, and the total ignorance of the pseudo-dentist who has crept into the profession, without the knowledge of the first principles, either of its theory or practice. In such cases, our duty, as the guardians of the teeth, require us to condemn, in the strongest terms, both the operator and his operations. A system of petty warfare between members of the profession is productive of good to none, and is, by no means, a very harmless or consistent amusement. Yet not unlike most professions, ours is not entirely free from this blemish upon its character. This warfare is not so often carried on openly as covertly; and the weapons commonly used are impalpable, and not easily parried; and consists mostly of hints and inuendos. An example will illustrate my meaning: A person who has had a tooth extracted at your office, a week or two previously, enters mine, with the idea that his jaw has been fractured, or that portions of the root still remain. I remove a slight fragment of the alveolar process, and in answer to his inquiry of "what is it?" I reply, "*A piece of the jaw bone!*" This assertion being unqualified, fully confirms the patient in his belief that the tooth had not been properly extracted. Again; you may be so unfortunate as to break off the crown of the cuspidate, and your patient will not allow you to complete the operation, but comes to my office, some time after when it has become loosened in the socket, and I remove it so easily, that the patient can hardly believe the evidence of his senses, when he sees the long, bloody root in his hand. In reply to his look of wonder and astonishment, I carelessly say, "O that's nothing, when you know *how* to do it!" My design might not be to injure a member of the profession, but to elevate myself in the estimation of the patient and of whomsoever he might tell of my wonderful performance; yet I wrong you by implication. The man who adopts such a mode of practice, or a similar one in regard to *any* of the various operations of other dentists, cannot but be regarded as a dishonest and dishonorable member of the profession. When our patients have come from another's office, we should use the utmost candor, under all circumstances, and be particularly cautious to use no deceit or evasion that will tend to injure *unjustly* our profession; at the same time, having the good of our patient eminently in view. This is imperatively demanded, both by considerations of *duty* and *interest.*

The man that is skillful, honest, and industrious, and devotes the best energies of his life to the practice of a useful profession, should receive a liberal remuneration for his services, so that, when he becomes disqualified by the infirmities of age, he may be able to live, if not in affluence, at least comfortably and independently, during the remainder of his life. In order to secure this, the dentist should be better paid *for the time he employs*, than are those engaged, in most other callings, and especially those engaged in commercial pursuits. For *they*, in their declining years are generally the most prosperous ; and often, in the afternoon of their life, reap their most abundant harvest of wealth. While at the same period in *his* life, the dentist becomes unfitted for the skillful performance of his professional duties ; for, although his mental vigor may remain unimpaired, his right hand has forgotten its cunning ; the firm, steady, yet delicate touch, so necessary to his manipulations, has become feeble and uncertain, his strained and over-taxed eye-sight has also become prematurely weakened and dulled : and now it is too late for him to engage in other business pursuits, with a probable prospect of success. Hence, I say, the dentist who has given the vigor of his life to his profesion *should be more liberally compensated for his skill and time employed* than are men engaged in most other professions and pursuits. This important fact is not sufficiently impressed upon the minds of our employers, nor on the minds of those engaged in the practice of our profession. And while I would not contend that the dentist, for his *thirty* years of practice, should receive an amount equal to that which some other professional men, and men engaged in trade, realize for their labors of half a century, yet I do most conscientiously hold that *his* compensation, in proportion to *theirs*, should be much greater than *thirty* is to *fifty*.

The subject of compensation for professional services between the dentist and his patrons, is often a delicate and embarrassing one, as there can be no fixed standard of their value. They depend upon the skill of the operator, the difficulty of the operation, the amount of time and material used, the benefit rendered, and, upon the ability of the patient. And, while there may be, sometimes, cases, in which it is the duty —or privilege of the dentist to work for a small and insufficient remuneration, or even nothing,—he owes it to his profession, as well as to himself, that when his patient has the ability, *his services should be recompensed according to a liberal standard.*

And now, gentlemen, having only glanced at what appeared to me to be the most prominent features of the subject under consideration, I will simply add, in conclusion, that, while we study to become useful and eminent in our profession, and labor for its elevation, honor, and advancement, we should be careful, not to become mere dentists. For, to reach the higher walk of a profession, something more is needed. Let the collateral sciences and polite literature, be cultivated, in our hours of relaxation. Not so much for their practical utility, as for their mental discipline, and rich stores of pleasure and knowledge which they afford. For there is great danger that, in the every day routine of our profession, to the exlusion of every thing else, the mind will become cramped and dwarfed, so as to disable it from taking enlarged and comprehensive views of topics which lie beyond the reach of our professional domain. And there is nothing so well adapted to fill up the interstices of business, to afford us rational enjoyment, and to smooth pleasantly, and honorably, the downward path of age.

